# Mortality, morbidity and educational outcomes in children of consanguineous parents in the Born in Bradford cohort

**DOI:** 10.12688/wellcomeopenres.22547.2

**Published:** 2024-09-26

**Authors:** Neil Small, Brian Kelly, Daniel S. Malawsky, Rajib Lodh, Sam Oddie, John Wright

**Affiliations:** 1 Faculty of Health Studies, University of Bradford, Bradford, England, UK; 2 Bradford Institute for Health Research, Bradford, UK; 3 Wellcome Sanger Institute, Hinxton, England, UK; 4 Bradford Teaching Hospitals NHS Foundation Trust, Bradford, England, UK

**Keywords:** Consanguinity, cohort study, health outcomes, education outcomes

## Abstract

**Background:**

Children of consanguineous parents have a higher risk of infant and childhood mortality, morbidity and intellectual and developmental disability.

**Methods:**

Using a UK based longitudinal cohort study we quantify differences according to the consanguinity status of children from birth to 10 in mortality, health care usage, two health and three educational outcomes. The cohort comprises 13727 children; 35.7% White British, 43.7% Pakistani heritage, and 20.8% are from other ethnic groups.

**Results:**

Compared to children whose parents were not related children whose parents were first cousins were more likely to die by age 10 (odds ratio 2.81, 95% CI 1.82-4.35) to have higher rates of primary care appointments (incident rate ratio 1.39, 95% CI 1.34-1.45) and more prescriptions (incident rate ratio 1.61, 95% CI 1.50-1.73). Rates of hospital accident and emergency attendance (incident rate ratio 1.21,95% CI 1.12-1.30) and hospital outpatients’ appointments (incident rate ratio 2.21,95% CI 1.90-2.56) are higher. Children of first cousins have higher rates of speech/ language development difficulties (odds ratio 1.63, 95% CI 1.36-1.96) and learning difficulties (odds ratio 1.89, 95% CI 1.28-2.81). When they begin school they are less likely to reach phonics standards (odds ratio 0.73, 95% CI 0.63-0.84) and less likely to show a good level of development (odds ratio 0.61, 95% CI 0.54-0.68). At age 10 there are higher numbers with special educational needs from first cousin unions compared to all children whose parents are not blood relations (odds ratio 1.38, 95% CI 1.20-1.58). Effect sizes for consanguinity status are similar in univariable and multivariable models where a range of control variables are added.

**Conclusions:**

There is higher childhood mortality and greater use of health care as well as higher rates of learning difficulties, speech and language development challenges and substantive differences in education outcomes in children whose parents are first cousins.

## Introduction

Consanguinity is a term generally used to describe parents who are blood-related individuals who share a recent common ancestor, for example first cousin unions are when both partners share a grandparent and second cousin unions share a great-grandparent. Consanguineous unions are considered common when a country has rates above 20% ( Bittles, 2012). More than one billion people worldwide live in societies where consanguineous marriages are common. Overall, in the UK consanguinity rates are low but it is common in some communities ( Small *et al.*, 2024a).

Worldwide, the published literature indicating an increase in infant and childhood morbidity in the children of consanguineous couples is extensive, as is a recognition of deaths in infancy being higher in children of first cousin unions when compared with non-consanguineous couples ( Bittles, 2012, 136; Bittles & Black, 2010). Malawsky *et al.* (2023) and Clark *et al.* (2019) report an impact of consanguinity across a range of common illnesses and health-related traits (body mass index, blood pressure, blood traits) across the life-course. A contributory role for consanguinity in childhood intellectual and development disability has been apparent for a considerable time ( Bittles, 2012: 152; Gidziela *et al.*, 2023; Gustavson, 2005) and an increase in reaction time (a correlate of general cognitive ability) and reduced educational attainment was reported in Clark *et al.* (2019).

In this paper we examine all-cause mortality and morbidity and selected education outcomes in children up to age 10 from Born in Bradford (BiB) to identify differences between children born to consanguineous parents and those whose parents are not related by blood.

## Methods

### Setting

Between 12 ^th^ March 2007 and 24 ^th^ December 2010 BiB, an ongoing birth cohort study based in the city of Bradford in the north of England, collected detailed information from 12453 women with 13776 pregnancies (in the recruitment years some women had more than one pregnancy) and from 3448 of their partners. All the recruited women were under the care of the Bradford Royal Infirmary and were in or near the 28 ^th^ week of their pregnancy (see Raynor & Born in Bradford Collaborative Group, 2008 for the study protocol and Wright *et al.*, 2013 to see cohort characteristics). Bradford is the sixth largest city in the UK with a population of about half a million and has urban areas that are among the most deprived in the UK. Sixty percent of the babies born in the city are born into the poorest 20% of the population of England and Wales based on the British government’s residential area Index of Multiple Deprivation.

### Data: consanguinity exposure measure and other covariates

Self-reported consanguinity status was collected as part of a wide-ranging interviewer administered questionnaire at recruitment to BiB. A section of this questionnaire asked whether the woman was related to the father of their baby, and if they answered “yes” they were then asked in what way they were related with the options in the questionnaire being; ‘First cousin’, ‘First cousin once removed’, ‘Second cousin’ and ‘Other related by blood’. The answers to these two questions were used to construct three categories of consanguinity; children whose parents were not blood related (‘not related’), children whose parents were first cousins (‘first cousins’), and children whose parents were other blood relations (‘other blood relations’).

The questionnaire also captured a number of covariates that we have used in this analysis: women’s age, educational status, and whether the household was in receipt of means tested state benefits. In the UK, being in receipt of means-tested benefits is recognised as a measure of income poverty ( Platt, 2007). The education status of women educated outside the UK were equivalised to UK levels and grouped to a dichotomous measure of A-level or above and below A-level. Achieving A-level or above requires continuing in education post age 16 years, and the division between those who stay and those who finish education has been identified as a key measure of educational inequalities ( Tackey *et al.*, 2011). Women recruited to the study gave consent to link their child’s routine healthcare data and education data, and from birth records we obtained the child’s gender, birthweight and gestational age at birth.

**Figure 1.**
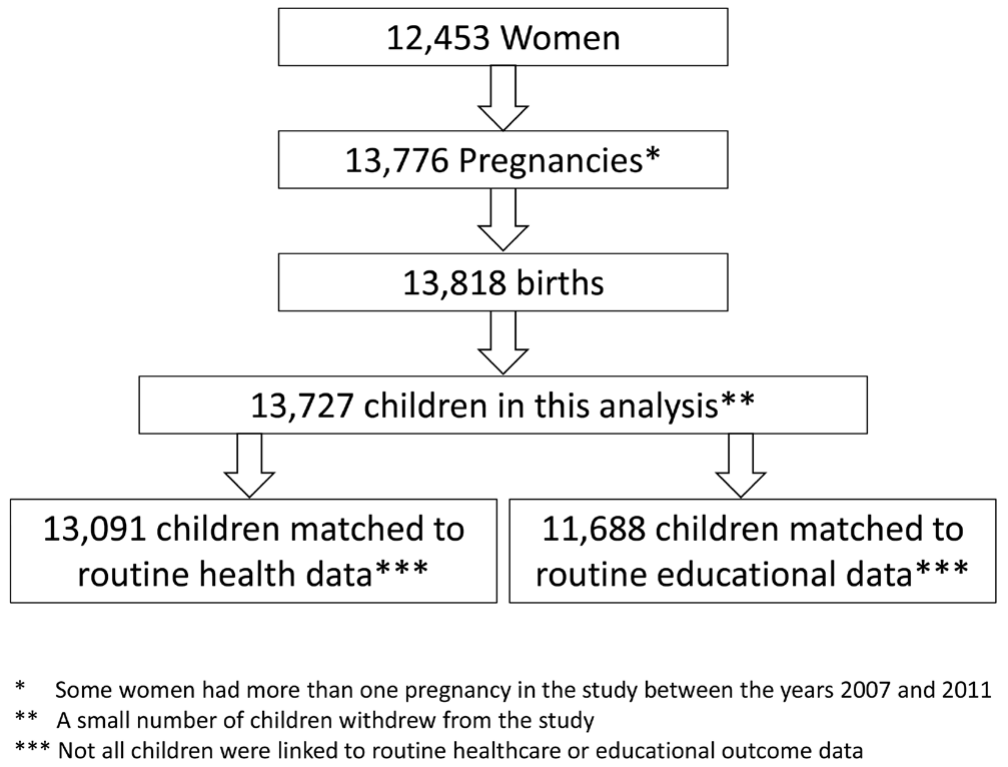
Number of women, pregnancies, children included in the analysis and children matched to routine healthcare data and educational outcomes data.

In total there were 13,818 children in the BiB cohort. A small number of children withdrew from the study, leaving 13,727 children included in the analysis, 13,091 of these children were matched to routine healthcare data and 11,688 were matched to educational outcomes data (see Figure 1).

### Data: outcomes

Mortality to age 10 is reported using routine NHS data and morbidity is considered in two ways; first as reflected in health care usage in general practice and hospital care, and second in two specific areas where there is putative evidence of a link with consanguinity, learning disability and speech and language development difficulties. Educational outcomes span the first years BiB children are in school: Early Years Foundation Stage (EYFS) assessment when children go to school (aged 4 to 5), phonics at Year 1 and special educational needs status at Year 6 (aged around 10 years).

Child health outcomes were determined at age 10 years (all counts of events up to the age of 10 years, or presence of conditions as at 10 years of age). Routine primary care data was obtained from Systmone ( https://tpp-uk.com/products/) which covers around a third of all primary care practices in England but all practices in Bradford. In total 95.4% of children were matched to primary care data, as indicated in Figure 1. Most children had a full ten years of linked routine data, with around 11% having less due to residential mobility (moving out of Bradford). The length of time that children were matched to routine primary care records was calculated, mean of 9.86 years (standard deviation of 1.07 years), and this was used as a measure of exposure.

A number of health outcomes were derived from linked routine healthcare primary care records: child deaths, the number of primary care appointments and prescriptions, the number of accident and emergency hospital events and outpatient hospital events, the presence of a diagnosis of learning difficulties, and of a diagnosis of speech and language difficulties in the primary care records. Child deaths consisted of both full-term stillbirths and death from any cause up to age 10 years, both these events being recorded in routine data. The count of appointments was derived from clinical Read codes (i.e., after removing all non-clinical Read coded events). Read codes are used to code elements of each primary care appointment, there can be one or many Read codes associated with each appointment, (for further details of Read codes see: https://digital.nhs.uk/services/terminology-and-classifications/read-codes). The routine primary care data also contains a record of every medicine prescribed. Prescriptions are recorded using the British National Formulary (BNF) coding system ( https://bnf.nice.org.uk/). We counted the number of individual appointments and prescriptions for each child up to the age of 10 years. Hospital accident and emergency and outpatient events were identified from records in primary care (any hospital event is notified and recorded here including those outside Bradford). A search for hospital event related Read codes was made by searching the text of Read code descriptions, and the Read codes identified were then classified as either relating to accident and emergency or outpatient events. Counts of these events for each child were calculated up to age 10 years. We also used Read codes to identify the presence of learning difficulties, and speech and language development difficulties. (See Additional Analysis 1 in Small *et al.*, 2024b for further details.)

Educational data was obtained from the local authority education department at the City of Bradford Metropolitan District Council. A number of educational outcomes were used in the analysis. We looked at the Early Years Foundation Profile (EYFP) results for children, this measures learning and development of children at around five years of age ( https://www.gov.uk/early-years-foundation-stage). We used the dichotomous measure of whether or not a child had reached a 'good stage of development' in the assessment. We also identified whether the child had achieved the required level of phonics understanding. Phonics is a way to teach children to read through learning sounds and is taught in a structured way, starting with the easiest sounds and progressing through to the most complex, it is widely believed to be the most effective way of teaching young children to read, and as being particularly helpful for children aged five to six years of age. ( https://www.gov.uk/education/phonics). Finally, we identified whether children had been recorded as having special educational needs status (SEN). Section 20 of the UK Children and Families Act 2014 ( https://www.legislation.gov.uk/ukpga/2014/6/part/3/enacted) defines a child as having special educational needs (SEN) if he or she "has a learning difficulty or disability which calls for special education provision to be made for him or her". A child is considered to have special educational needs if she or he has a significantly greater difficulty in learning than the majority of others of the same age; or has a disability which prevents or hinders them from making use of facilities of a kind generally provided for others of the same age in mainstream schools. We searched the educational records for children who had a classification of SEN by school year 6 (where children are aged around 10 or 11 years).

### Plan of analysis

The analysis was carried out at the child level. As there was a separate questionnaire completed at each pregnancy we measured the child’s parental consanguinity status even if the mother had multiple pregnancies in the study and mother’s partner changed over time. In the analysis we first present a profile of the cohort with descriptive statistics detailing self-reported consanguinity status, child gender, child low birthweight and pre-term births, maternal education status, mother's age at birth of the child, and household means-tested benefit status. We then present descriptive statistics of cohort characteristics by consanguinity status, and finally descriptive analysis of the health and education outcome measures. We then employed a series of regression models to estimate odds ratios and incident rate ratios, as well as predicted rates and probabilities, for each child health and education outcome. We estimated separate univariable and multivariable models. The univariable models contained only the covariates of consanguinity status, and the multivariable models additionally controlled for the cohort characteristics outlined above. We employed logistic regression for dichotomous outcomes (whether the child died, diagnosis of learning difficulties, diagnosis of speech and language difficulties, reaching good stage of development in school reception year, achieving phonics standard by school year 1, being recorded as having special educational need by age 10 years), and Poisson regression for counts of healthcare use events (primary care appointments, primary care prescriptions, hospital accident and emergency events, hospital outpatient events). We estimated odds ratios for dichotomous outcomes and incident rate ratios for counts of events. In addition to odds ratios and incident rate ratios we also calculated marginal effects ( Williams, 2012) to derive predicted probabilities and predicted rates. In all regression models we dealt with missing data by carrying out a complete case analysis. All statistical analysis was carried out using Stata 17 ( StataCorp, 2023).

We present tables of odds ratios and incident rate ratios, and figures of predicted probabilities and predicted rates from multivariable models for all outcomes. After the main analysis we present a sensitivity analysis for differences in outcomes using genetically derived consanguinity status of parents in a subset of the cohort. We also present a sensitivity analysis considering results for Pakistani heritage children compared to all children.

## Results

### 1. Cohort characteristics

Table 1 shows the cohort characteristics. Most children, 72.0%, had parents who self-reported as being not related, 17.7% of parents were first cousins, and 10.3% were other blood relations. Table 1 also shows that 51.6% of children were male and 48.4% were female, 8.8% were low birthweight (less than 2500 grams), 6.7% were born pre-term (less than 37 weeks), 43.7% of the children’s mothers were educated to A-level or above, and 40.9% were in receipt of means-tested benefits. The majority of mothers in the study were born in England (7038: 62.5%), 2887 mothers (25.6%) were born in Pakistan. The remainder were born in a wide range of countries with no one country providing more than 1.5% of mothers. In our study population, recruited between 2007 and 2010, over 90% of children whose parents were first cousins or other blood relations were of Pakistani heritage. Rates of consanguinity amongst the population in Bradford have fallen substantially over recent years ( Small *et al.*, 2024a). Levels of missing data are lower for measures derived from linked routine birth outcome data; there were higher levels of missing data for measures derived from the BiB maternal baseline questionnaire, as not all women completed this questionnaire at recruitment.

**Table 1.** Cohort characteristics.

Cohort characteristics	N	Percentage
Consanguinity status of child’s parents		
Not related	8056	72.0%
First cousin	1977	17.7%
Other blood relation	1152	10.3%
Missing	2542	
Total	13727	100.0%
Child gender		
Male	6992	51.6%
Female	6561	48.4%
Missing	174	
Total	13727	100.0%
Child's ethnicity		
White British	4857	35.7%
Pakistani heritage	5920	43.5%
Other ethnicity	2838	20.8%
Missing	112	
Total	13727	100.0%
Mother's country of birth		
England	7038	62.5%
Northern Ireland	21	0.2%
Scotland	37	0.3%
Wales	20	0.2%
Channel Islands	2	0.0%
Isle of Man	3	0.0%
Republic of Ireland	7	0.1%
Czech Republic	16	0.1%
Poland	174	1.5%
Slovakia	19	0.2%
Bangladesh	151	1.3%
India	221	2.0%
Pakistan	2882	25.6%
Sri Lanka	1	0.0%
Philippines	66	0.6%
Other	610	5.4%
Missing	2459	
Total	13727	100.0%
Child birthweight (low birthweight = less than 2500g)		
Not low birthweight	12068	91.2%
Low birthweight	1164	8.8%
Missing	495	
Total	13727	100.0%
Child gestational age at birth (pre-term birth = before 37 weeks)		
Not pre-term birth	12353	93.3%
Pre-term birth	881	6.7%
Missing	493	
Total	13727	100.0%
Mother's educational status		
A-level or higher	4517	43.5%
Lower than A-level	5861	56.5%
Missing	3349	
Total	13727	100.0%
Mother's age at birth of child		
15 to 20 years	1396	11.3%
21 to 24 years	2986	24.2%
25 to 29 years	3595	29.1%
30 to 34 years	2651	21.5%
35 to 49 years	1710	13.9%
Missing	1389	
Total	13727	100.0%
Household means-tested benefit status		
In receipt of means-tested benefits	4595	40.9%
Not in receipt of means-tested benefits	6639	59.1%
Missing	2493	
Total	13727	100.0%

Table 2 looks at the association between consanguinity status and the other cohort characteristics. There were differences in rates of low birthweight, levels of maternal education, and differences in the proportion of households in receipt of means tested benefits between children with parents of different consanguinity status. We found 12.2% of children whose parents were first cousins had a low birthweight compared to 7.6% of children whose parents were not related, 31.0% of mothers who were first cousins of their partner were educated to A-level or above compared to 48.0% of mothers who were not related to their partner, and 49.5% of children whose parents were first cousins lived in households in receipt Data section). of means tested benefit compared to 37.3% of children whose parents were not related.

**Table 2.** Cohort characteristics of children by parental consanguinity status (excludes 2542 children with missing data on parental consanguinity status).

	Consanguinity status of child's parents						
	Not related		First cousin		Other blood relation		p value (from Chi square test)
	n	%	n	%	n	%	
Child ethnicity							
White British	4198	52.1%	10	0.5%	8	0.7%	
Pakistani heritage	1965	24.4%	1812	91.7%	1057	91.8%	
Other ethnicity	1887	23.4%	155	7.8%	87	7.6%	
Missing	6		0		0		
Total	8056	100.0%	1977	100.0%	1152	100.0%	< .001
Child gender							
Male	4113	51.5%	1008	51.3%	580	50.5%	
Female	3879	48.5%	956	48.7%	568	49.5%	
Missing	64		13		4		
Total	8056	100.0%	1977	100.0%	1152	100.0%	.837
Child birthweight (low birthweight = less than 2500g)							
Not low birthweight	7171	92.4%	1708	87.8%	1023	90.5%	
Low birthweight	590	7.6%	237	12.2%	107	9.5%	
Missing	295		32		22		
Total	8056	100.0%	1977	100.0%	1152	100.0%	< .001
Child gestational age at birth (pre-term birth = before 37 weeks)							
Not pre-term birth	7253	93.4%	1819	93.5%	1067	94.4%	
Pre-term birth	510	6.6%	126	6.5%	63	5.6%	
Missing	293		32		22		
Total	8056	100.0%	1977	100.0%	1152	100.0%	.445
Mother's educational status							
A-level or higher	3518	48.0%	579	31.0%	393	35.7%	
Lower than A-level	3812	52.0%	1286	69.0%	708	64.3%	
Missing	726		112		51		
Total	8056	100.0%	1977	100.0%	1152	100.0%	< .001
Mother's age at birth of child							
15 to 20 years	952	13.1%	113	6.3%	67	6.5%	
21 to 24 years	1646	22.7%	487	27.2%	285	27.5%	
25 to 29 years	2022	27.9%	570	31.8%	315	30.4%	
30 to 34 years	1584	21.8%	391	21.8%	233	22.5%	`
35 to 49 years	1056	14.5%	232	12.9%	137	13.2%	
Missing	796		184		115		
Total	8056	100.0%	1977	100.0%	1152	100.0%	< .001
Household means-tested benefit status							
In receipt	2997	37.3%	975	49.5%	576	50.1%	
Not in receipt	5034	62.7%	995	50.5%	574	49.9%	
Missing	25		7		2		
Total	8056	100.0%	1977	100.0%	1152	100.0%	< .001

### 2. Health and educational outcomes

Descriptive statistics of the health and educational outcomes are shown in Table 3a (for counts of health-related events) and Table 3b (for dichotomous health and education outcomes).

**Table 3a.** Health outcome counts (number of events in ten-year period).

Health outcomes (number of events in 10-year period)	n	Mean	Standard Deviation	Range	Inter Quartile Range
Primary care appointments	13091	33.6	25.0	0–328	17–44
Primary care prescriptions	13091	52.5	81.1	0–1837	14–59
Hospital accident and emergency events	13091	1.72	2.39	0–39	0–2
Hospital outpatient events	13091	1.23	3.89	0–101	0-1

**Table 3b.** Health and educational outcomes (dichotomous outcomes).

Health and education outcomes (dichotomous outcomes)	N	Percentage
Whether child died by age 10 *		
Yes	172	1.3%
No	13555	98.7%
Missing	0	
Total	13727	100.0%
Whether child diagnosed with learning difficulties by age 10		
Yes	208	1.6%
No	12883	98.4%
Missing	636	
Total	13727	100.0%
Whether child diagnosed with speech/ language difficulties by age 10		
Yes	1099	8.4%
No	11992	91.6%
Missing	636	
Total	13727	100.0%
Whether child reached good stage of development by school reception year **		
Yes	6675	59.4%
No	4565	40.6%
Missing	2487	
Total	13727	100.0%
Whether child achieved required level of phonics understanding by school year 1 ***		
Yes	8510	77.2%
No	2519	22.8%
Missing	2698	
Total	13727	100.0%
Whether child categorised as special educational needs status (SEN) by school year 6 ****		
Yes	2358	20.6%
No	9073	79.4%
Missing	2296	
Total	13727	100.0%

* Of those children that died most (148 of the 172) were stillbirths or aged under 1 years of age, only ten children died above age 3 years ** In England school reception year equates to children aged 4 to 5 years old *** In England school year 1 equates to children aged 5 to 6 years old **** In England school year 6 equates to children aged 10 to 11 years old

As Table 3a indicates, all counts of health outcome events (primary care appointments and prescriptions, and hospital events) were highly skewed; with some children having counts far greater than the mean or interquartile range. This reflects the needs of a small minority of children who have more serious health conditions. The mean number of primary care appointments was 33.6 in the ten-year period (i.e., just over three a year); but 1,160 children (around 9%) had double the mean number of appointments or more, and 299 children (around 2%) had 100 appointments or more. As demonstrated by the interquartile range, half of children had between 17 and 44 primary care appointments in the ten-year period. The distribution of the number of prescriptions was similar; the mean number was 52.5, the interquartile range was 14 to 59 prescriptions in the ten-year period. A small number of children had very high numbers of prescriptions, 1,689 (12.9%) had 100 or more prescriptions, 225 (1.7%) had 300 or more prescriptions, and 8 children had more than 1,000 prescriptions. Hospital related events occurred much less frequently; the mean number of accident and emergency or outpatient events was less than 3 in the ten-year period. Just over a fifth of children (23.5%) had no accident and emergency events, and only 4.2% had 10 or more.

Table 3b illustrates the dichotomous health and educational outcomes. A total of 172 (1.3%) of children had died by the age of 10 years, mostly at birth or in the first year after birth. By the age of 10 years 208 (1.6%) were diagnosed with learning difficulties. A learning difficulty is defined by the Department of Health and Social Care (DHSC), 2001 as: “a significantly reduced ability to understand new or complex information, to learn new skills (impaired intelligence), with a reduced ability to cope independently (impaired social functioning), which started before adulthood.1099 (8.4%) children had been diagnosed with speech or language difficulties. These are termed “developmental language disorders” and defined as a communication disorder that interferes with learning, understanding, and using language. These language difficulties are not explained by other conditions, such as hearing loss or autism, or by extenuating circumstances, such as lack of exposure to language ( NIDCD, 2024). A substantial number of children, 4565 (40.6%), had not reached a good stage of educational development by the end of reception year, aged 4 to 5 years; and 2519 (22.8%) had not reached the required level of phonics by the end of school year one, aged 5 to 6 years. Also 2,358 (20.6%) children were classified as having special educational needs by school year six when they were aged 10 to 11 years.

### 3. Regression models exploring health and educational outcomes by consanguinity status

**Figure 2.**
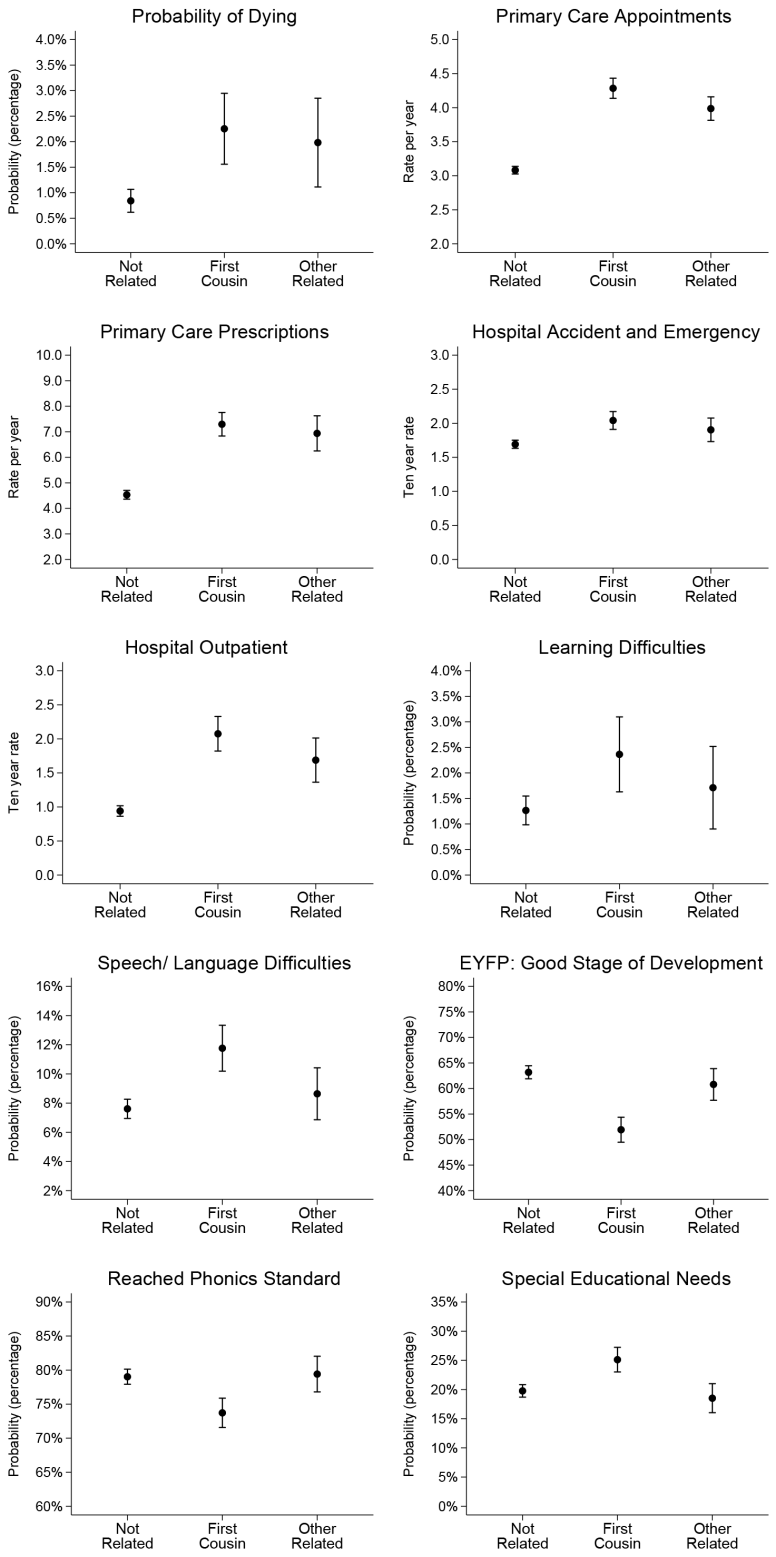
Predicted probability and predicted rates for health and education outcomes by consanguinity status from multivariable models. For full results of predicted probabilities and rates from univariable and multivariable models see Small *et al.*, 2024b.

We explored health and educational outcomes in separate univariable models by consanguinity status of the child’s parents, then in separate multivariable models; controlling for child gender, low birthweight, pre-term birth, mother’s education status, mother’s age at birth of the child, and whether the household was in receipt of means-tested benefits. Results from the multivariable models are reported as odds ratios and incident rate ratios in Table 4, with the full results from univariable and multivariable models given in Additional Analysis 2 ( Small *et al.*, 2024b). Effect sizes for consanguinity status are similar in the univariate and multivariable models, the latter with the control variables added. This suggests that the effect of consanguinity status is largely independent of other variables in the models that are considered in the academic literature to lead to poor health and to impact on educational outcomes. Predicted probabilities and predicted rates for all outcomes from the multivariable models are illustrated in Figure 2, these results are reported in tables in Additional Analysis ( Small *et al.*, 2024b).

**Table 4.** Odds ratios/ incident rate ratios form multivariable regression models with 95% confidence intervals from multivariable models (reference group = not related).

	Reference group = not related			
	First cousin		Other blood relation	
Health and Educational Outcomes	Ratio	(95% CI)	Ratio	(95% CI)
Whether died	2.81	(1.82-4.35)	2.45	(1.42-4.24)
Primary care appointments	1.39	(1.34-1.45)	1.29	(1.23-1.36)
Primary care prescriptions	1.61	(1.50-1.73)	1.53	(1.38-1.70)
Hospital accident and emergency events	1.21	(1.12-1.30)	1.13	(1.02-1.24)
Hospital outpatient events	2.21	(1.90-2.56)	1.80	(1.46-2.21)
Learning difficulties	1.89	(1.28-2.81)	1.36	(0.80-2.32)
Speech and language development difficulties	1.63	(1.36-1.96)	1.15	(0.90-1.48)
Early years foundation profile: good stage of development	0.61	(0.54-0.69)	0.90	(0.77-1.04)
Phonics standard	0.73	(0.64-0.84)	1.03	(0.85-1.23)
Special educational needs status	1.38	(1.20-1.58)	0.92	(0.76-1.11)

For full results of odds ratios/ incident rate ratios from univariable and multivariable models see Small *et al.*, 2024a.

***3.1 Child deaths.*** From the multivariable regression models, we found that children whose parents were first cousins had a much greater probability of dying by the age of 10 years compared to children whose parents were not related (odds ratio 2.81, 95% CI 1.82-4.35). Figure 2 illustrates the predicted probability of dying by the age of 10 years was 1.28% (95% CI: 0.69%-1.87%) for children whose parents were first cousins, compared to 0.57% (95% CI: 0.00%-1.20%) for children whose parents were other blood relations, and 0.21% (95% CI: 0.06%-0.35%) for children whose parents were not related.

***3.2 Rates of healthcare usage.*** In general children whose parents were first cousins, and to a lesser extent children whose parents were other blood relations, had higher rates of healthcare use compared to children whose parents were not related.

In the multivariable models there were substantial differences in rates of primary healthcare use and hospital events, particularly outpatient hospital events, by the child’s parental consanguinity status. Results of the multivariable Poisson regression models reported in Table 4 shows that children whose parents were first cousins had around 39% higher incidence of primary care appointments, and 61% higher incidence of prescriptions compared to children whose parents were not related: incident rate ratio of 1.39 (95% CI 1.34-1.45) and 1.61 (95% CI 1.50-1.73) respectively. Also, children whose parents were first cousins had around 21% higher incidence of hospital accident and emergency events and over twice the rate of hospital outpatient events compared to children whose parents were not related: incident rate ratio of 1.21 (95% CI 1.12-1.30) and 2.21 (95% CI 1.90-2.56) respectively.

Figure 2 illustrates that the predicted rate of primary care appointments per year was 4.13 (95% CI: 3.98-4.28) for children whose parents were first cousins, compared to 3.73 (95% CI: 3.52-3.93) for children whose parents were other blood relations, and 3.00 (95% CI: 2.93-3.07) for children whose parents were not related. Predicted rates of primary care prescriptions per year were 6.82 (95% CI: 6.35-7.28) for children whose parents were first cousins, compared to 6.01 (95% CI: 5.44-6.58) for children whose parents were other blood relations, and 4.32 (95% CI: 4.10-4.54) for children whose parents were not related. The ten year rate of accident and emergency hospital events was 2.00 (95% CI: 1.86-2.14) for children whose parents were first cousins, compared to 1.70 (95% CI: 1.48-1.91) for children whose parents were other blood relations, and 1.66 (95% CI: 1.59-1.74) for children whose parents were not related. The ten year rate of outpatient hospital events was 1.98 (95% CI: 1.71-2.24) for children whose parents were first cousins, compared to 1.43 (95% CI: 1.10-1.76) for children whose parents were other blood relations, and 0.85 (95% CI: 0.76-0.94) for children whose parents were not related.

***3.3 Specific health conditions: learning difficulties, and speech and language development difficulties.*** We describe substantial differences in the probability of a child being diagnosed with learning difficulties and speech and language development difficulties between children whose parents were first cousins compared to children whose parents were not related. The estimated differences between children whose parents were other blood relations were not different from children whose parents were not related (considering the 95% confidence intervals around the estimates).

Table 4 shows that children whose parents were first cousins were 89% more likely to be diagnosed with learning difficulties and 63% more likely to be diagnosed with a speech and language development difficulty compared to children whose parents were not related; odds ratio 1.89 (95% CI: 1.28-2.81) and 1.63 (95% CI: 1.36-1.96) respectively.

Figure 2 illustrates that the probability of being diagnosed with learning difficulties was 2.28% (95% CI: 1.53%-3.03%) for children whose parents were first cousins, compared to 1.06% (95% CI: 0.22%-1.90%) for children whose parents were other blood relations, and 1.01% (95% CI: 0.68%-1.33%) for children whose parents were not related. The probability of being diagnosed with speech and language learning difficulties was 11.3% (95% CI: 9.6%-12.9%) for children whose parents were first cousins, compared to 6.40% (95% CI: 4.33%-8.46%) for children whose parents were other blood relations, and 7.35% (95% CI: 6.51%-8.18%) for children whose parents were not related.

***3.4 Educational outcomes.*** There are substantial differences in the probability of a children having poor educational outcomes between children whose parents were first cousins compared to children whose parents were not related. But the estimated differences between children whose parents were other blood relations were not different from children whose parents were not related (considering the 95% confidence intervals around the estimates).

Table 4 shows that children whose parents were first cousins were less likely to reach a good stage of development in the Early Years’ Foundation Profile at age 4 to 5 years, and less likely to reach the phonics standard at age 5 to 6 years compared to children whose parents were not related; odds ratio 0.61 (95% CI: 0.54-0.69) and 0.73 (95% CI: 0.64-0.84) respectively. Children whose parents were first cousins were more likely to be recorded as having special educational needs by age 10 to 11 years; odds ratio 1.38 (95% CI: 1.20-1.58).

Figure 2 illustrates that the probability of reaching a good stage of development in the early years’ foundation profile was 53.7% (95% CI: 51.0%-56.3%) for children whose parents were first cousins, compared to 58.4% (95% CI: 54.2%-62.7%) for children whose parents were other blood relations, and 64.0% (95% CI: 62.4%-65.7%) for children whose parents were not related. The probability of achieving the phonics standard was 74.4% (95% CI: 72.1%-76.7%) for children whose parents were first cousins, compared to 79.5% (95% CI: 75.9%-83.1%) for children whose parents were other blood relations, and 79.4% (95% CI: 78.0%-80.8%) for children whose parents were not related. The probability of being recorded as having special educational needs was 22.3% (95% CI: 20.1%-24.5%) for children whose parents were first cousins, compared to 19.5% (95% CI: 16.0%-23.0%) for children whose parents were other blood relations, and 19.0% (95% CI: 17.6%-20.3%) for children whose parents were not related.

### 4. Sensitivity analysis

***4.1 Sensitivity analysis using genetically derived consanguinity status for a subset of children.*** We carried out a sensitivity analysis using genetically derived consanguinity that was available for a subset of the Born in Bradford cohort, an approach reported in Arciero *et al.*, 2021. Using the patterns of homozygosity observed in a child’s genome, Arciero and colleagues developed a machine learning algorithm to infer the degree of relatedness of an individual’s biological parents. Genetically derived consanguinity status was stratified into three categories, having parents inferred to be first cousins or closer, first cousins once removed/second cousins, or further than second cousins (unrelated) These categories are comparable to the three categories of self-reported consanguinity used in BiB.

A total of 9158 children had DNA samples, around 60% of the children in the BiB cohort. Additional Analysis 3 ( Small *et al.*, 2024b) describes the genetically derived consanguinity measure and compares this to the self-reported measures. The ethnicity of the subset of children who had genetically derived consanguinity status was very similar to the ethnicity of the children in the sample using self-reported consanguinity. Just over a third (35.6%) were White British, 44.1% were Pakistani heritage, and 20.3% were from other ethnic groups. Rates of first cousin relationships are higher using the genetically derived consanguinity measure (24.3%) compared to self-reported consanguinity (17.7%). The self-reported and genetically derived first cousins are fairly similar (90.2% of self-reported first cousin relationships are also first cousins in the genetically derived measure). However, only around a third (34.7%) of those who have self-reported other blood relationships parents were inferred to have second cousins or closer parents in the genetically derived measure; over half (53.3%) of those who reported other blood relationships were first cousins in the genetically derived measure. There was substantial amount of missing data for the genetically derived consanguinity measure, there was also a smaller amount of missing data on self-reported consanguinity status (largely due to not all mothers of BiB children completing a baseline questionnaire). Of the 13727 children 2542 (18.5%) had missing self-reported consanguinity status, and 5457 (39.8%) had missing genetically derived consanguinity status. There are differences in the distribution of this missing data. In the cohort 172 children died by the age of 10 years; of these children 16.9% had missing data on self-reported consanguinity, but 76.2% had missing data on genetically derived consanguinity. In the cohort 881 children were born pre-term; 20.7% had missing data on self-reported consanguinity, but 54.5% had missing data on genetically derived consanguinity.

Because there were differences in consanguinity status of parents between the self-reported and genetically derived measures we re-ran regression models for differences in the health and education outcomes using the genetically derived consanguinity status measure, see Additional Analysis 4 ( Small *et al.*, 2024b). The differences in health and educational outcomes by consanguinity status were generally very similar whether the measure of consanguinity status was self-reported or genetically derived. However, there are differences between the self-reported and genetically derived measures of the probability of dying by the age of 10 years. As noted above there was more missing data for the genetically derived measure for children who have died. This is likely to explain differences in the probabilities of dying between the self-report and genetically derived measures.

***4.2 Sensitivity analysis considering results for Pakistani heritage children compared to all children.*** We have analysed the relationship between parental consanguinity status and child outcomes for all children in the Born in Bradford cohort. Our association of interest is between consanguinity and child outcomes. Many studies, including BiB studies, have looked at ethnicity and consanguinity, a focus that reflects very different rates of consanguinity observed between ethnic groups. In Bradford rates were highest in the parents of Pakistani heritage ( Small *et al.*, 2024a). We conducted a sensitivity analysis to look at the size of effects of consanguinity for all children compared to Pakistani heritage children, see Additional Analysis 5 in Small *et al.*, 2024b. The effects of consanguinity on child health and education outcomes are no different for Pakistani heritage children than they are for all children (considering 95% confidence intervals) for all outcomes apart from primary care appointments and prescriptions, where differences by consanguinity status were slightly larger for all children than for just Pakistani heritage children.

## Discussion

### The significance of CAs

We have previously identified consanguinity as a major risk factor for congenital anomalies in the BiB cohort. CAs occur in any births but consanguinity was associated with a doubling of risk for congenital anomaly in babies from first cousin unions. Babies whose parents were related by blood but were not first cousins were around 60% more likely than non-blood related parents to have an anomaly. Although risks of CA are lower in the non-consanguineous there are far more births in this category with the result that fifty two percent of BiB babies born with an anomaly did not have consanguineous parents ( Sheridan *et al.*, 2013).

Our 2013 study ( Sheridan *et al.*, 2013) identified 386 children in BiB as having a congenital anomaly, 3% of the total for whom data were available. Of those 201 had parents who were not consanguineous, 123 had parents who were in first cousin unions and 62 were related as “other blood” (second cousins). Bishop *et al.* (2017) linked children from BiB to a routine primary care database to detect CA diagnoses as the children grew older, from birth to age 5 years. Looking at a greater age range than Sheridan *et al.*, increased the ascertainment of children with CAs to 620.6 per 10000 live births in those under 5 years. In children under 5 primary care appointments, use of hospital services and referrals to specialists were higher for children with CA than those without ( Bishop *et al.*, 2018). As genetic diagnosis can be targeted to a specific gene, diagnosis is often undertaken antenatally, or in early life, for those who have been born to a family where there is already a child with a genetic condition. Consanguineous couples then may be more likely to have an early test. But subsequent detection of CAs and other genetic conditions will continue through childhood for children from both consanguineous and non-consanguineous unions.

We have reported that children who are from consanguineous unions have more hospital out-patient appointments, higher rates of learning difficulties, speech and language development challenges and they also exhibit differences in education outcomes. There is also an increased incidence of low birth weight babies, 12.2% in first cousins. 9.5% in “other blood” and 7.6% in non-related births ( Table 2). Being born low weight has its own adverse outcomes and as this is seen more frequently in this group, this is an additional negative health risk ( West *et al.*, 2018). Research on rare diseases in childhood using the BiB cohort has identified greater healthcare usage and an impact on education outcomes for a range of conditions including CAs and other genetic conditions, neurodegenerative disorders for example. This research found rare diseases distributed across the spectrum of backgrounds present in the cohort. It did not analyse their distribution by consanguinity status ( Lodh *et al.*, 2023).

Our health care usage data shows a highly skewed distribution with a relatively small group of children having considerably more primary care appointments and prescriptions – around 11% (1459 children) had twice the mean for appointments and 14.6% (1914 children) had a 100 or more prescriptions in a ten year period. The numbers represented in these higher healthcare usage groups are considerably greater than the number of children with CAs diagnosed by 5 years of age. There does then appear to be an additional more diffuse morbidity requiring the attention of primary care and hospital services associated with children whose parents were consanguineous, a diffusion consistent with the Clark *et al.* (2019), and Malawsky *et al.* (2023) results cited above, and consistent with the body of work Bittles (2012) refers to reporting links between consanguinity and a range of morbidities.

There are 32 countries in the world with consanguinity rates above 5% (according to The World Population Review - https://worldpopulationreview.com/country-rankings/inbreeding-by-country *accessed 1 Sept 2024*). The under 5 mortality rate for these countries ranges from 3.4 to 107.2 per 1,000 live births (according to the World Bank - https://data.worldbank.org/indicator/SH.DYN.MORT *accessed 1 Sept 2024*). Some countries with similar rates of consanguinity have vastly different under 5 mortality rates; for example, in the United Arab Emirates 50.5% of marriages are consanguineous and the under 5 mortality rate is 5.3 per 1,000 live births, while in South Sudan 50.0% of marriages are consanguineous and the under 5 mortality rate is 98.8 per 1,000 live births. Similarly in Tunisia 21.1% of marriages are consanguineous and the under 5 mortality rate is 11.5 per 1,000 live births, while in Nigeria 19.9% of marriages are consanguineous and the under 5 mortality rate is 107.2 per 1,000 live births. Countries vary greatly on levels of poverty and on the availability of healthcare provision, and these are the factors that impact most on child death rates.

### Recognizing the needs is an equity issue

The impact of consanguinity on mortality and morbidity in infancy and childhood in populations where consanguinity is commonplace should be considered in planning and providing health services. We have demonstrated increased use of services by children born to consanguineous parents in primary care, hospital care and in specialised education. Being cognizant of these patterns requires a response to what is a health care equity issue. So too is the need to inform and educate health care professionals about the breadth of impact consanguinity has on a health care ecology. Worldwide the WHO Global Burden of Disease resource recognises that the continuing care of the offspring of consanguineous unions is relevant for planning required levels and types of services ( Global Burden of Disease Collaborative Network, 2016). These worldwide demands are likely to increase as more children survive infancy.

There is also a need to consider the impact of the increased presence of educational challenges reported in children born from consanguineous unions. This is, like in the health differences we have reported, an equity and a planning and service provision issue. These children’s trajectories through education are likely to require focussed resources to tackle the different starting points they are at when they begin education so that they can fully realize their capacities, starting points that are impacted by social and biological factors (see respectively Cheung *et al.*, 2023 on the significance of deprivation in the frequency of late talking in 2 year olds in BiB and, as cited above, Clark *et al.*, 2019 on an increase in reaction time in children of consanguineous unions. This is a correlate of general cognitive ability.) In an education system where there are children who are likely to manifest these particular challenges school staff will benefit from understanding the significance of the findings we present as they plan schemes of learning in their classrooms and education providers need to accommodate these needs as they shape their budget allocations.

These levels of health care use and of educational outcomes interact in a way that can compound harm. The considerable amounts of time that some children will be away from school for treatment, or recovering from treatment, will be a factor in their reported educational outcomes. It is also likely that the demands of caring for a young child with CAs, or with other complex needs, will impact on parents, carers and on siblings ( Gimenez-Lozano *et al.*, 2022). Whole families are challenged by their having children with complex health and education needs ( Masefield *et al.*, 2022).

In addition to the resources required to achieve service equity there is also a health education and health promotion agenda to help make people aware of the impact of consanguinity throughout childhood as well as considering the increased risk of infant mortality. The agenda here should be to enhance informed choice about risks for children in populations where consanguinity is practised. There is a similar imperative to inform and educate about impacts on education.

Within the BiB cohort we have reported high levels of consanguinity compared to UK averages. Our recruitment to BiB occurred between 2007 and 2010 and since that time there has been a reduction in rates of consanguinity in the city ( Small *et al.*, 2024a). We have made available detailed research and routine data to illuminate the characteristics of the consanguineous and to follow up its impact on children. In so doing we have added to a growing international literature in which evidence is increasingly clear that, while there may be socio-economic benefits that contribute to the enduring practice of consanguinity ( Bhopal *et al.*, 2014), the evidence of wide-ranging harm is clear and convincing. It is also clear that promoting awareness and engagement of communities is best done with sensitivity to the cultural practices of those communities where consanguinity remains commonplace ( Darr *et al.*, 2013).

### On the sensitivity analysis

We have reported genetically derived consanguinity data for a subset of the BiB cohort and found a close match between self-identified and genetically identified first cousins but differences in those who self-identify as being in “other blood” relationships. Over half of those couples describing themselves as related but not first cousins appear to be first cousins on genetic analysis. This disparity may be a result of a lack of clarity in interviewees about what a first cousin and what “other-blood” is, it may be to do with people answering “other-blood” because they see a stigma attached to cousin-marriage and are seeking to mitigate this in the self-description they report to researchers. Sheridan *et al.* (2013) reported higher rates of CAs in the children of other blood unions (then identified through self-reporting) than were expected from a formal calculation of the relationship coefficient, a measure of genetic closeness ( Sheridan *et al.*, 2013: 8). This might be to do with endogamy, a longstanding tradition of consanguinity in a specific population allied with population stratification in marriage choices (see Bittles & Small, 2016; Small *et al.*, 2017; Woods *et al.*, 2006; Zlotogora & Shalev, 2010). In effect one can be akin to a cousin genetically, even if one is not a cousin in the familial sense. We will report separately on a qualitative study in Bradford contemporaneous with this one seeking views on the current importance of consanguinity in peoples’ choices of marriage partner.

We could assume that the genetically derived consanguinity measures would have less measurement error than the self-reported measures, therefore the size of observed effects would be larger. However, the results we report were essentially the same when using both measures apart from cases where the children with missing data were different. Given that children who have genetically derived data in BiB exclude many who died or were pre-term births, it may be that they could be considered as distinctly different samples. Therefore, the similarity of results using both measures could be seen as evidence of the robustness of the findings presented.

We have reported that the effects of consanguinity on child health and education outcomes are not different for Pakistani heritage children than they are for all children (considering 95% confidence intervals) for most outcomes. Our discussion of Pakistani heritage children is because they are the group with the highest rate of consanguinity in BiB but similar levels are likely to apparent in any ethnic, social or geographical group with similar proportions of consanguineous unions. Consanguinity is often approached via a concentration on specific ethnic groups where it is a more common practice. But our concern is to focus on its sequelae in terms of impact on health and education and not on its antecedent social structures. This approach frees a consideration of health and educational need from the baggage of an often fraught debate that too easily conflates a genetic and health risk with a cultural practice ( Darr *et al.*, 2016).

### Using a cohort study and routine data to look at health care usage

Although the study we report is in a single site it is a large and ongoing study with rich data sets enhanced by permission to access NHS and educational data relating to cohort members. These data allow us to include a wide range of possible confounders. Follow up rates in the cohort are high. The BiB study has been reporting on cohort members in a wide range of areas of interest and has accumulated extensive contextual insights into growing up in the city and considerable amounts of data relevant to key policy and practice domains ( www.borninbradford.nhs.uk). In consequence this study underlines the value of a long-running study accessing linked data in health systems to identify health care usage. It also illustrates the insights that can come from linking cohort data with school records. BiB data collection is ongoing and will, in the future, furnish insights into health care usage, health outcomes and educational attainment through adolescence and into adulthood ( Shire *et al.*, 2024). In doing this it will help address an absence of data on the effects of consanguinity on adult-onset diseases and on congenital anomalies that present in adulthood ( Bittles, 2013).

In the majority of this paper consanguinity is self-reported. Health care usage data and education data are from routinely collected sources. Health care usage has been used as a proxy for health outcomes, it does not capture all the complex morbidity people experience, its cumulative effect or its impact on individual lives. We have not looked at disease / pathology and hence we don’t know the mechanisms shaping the outcomes we report. We do not have data on social care usage for our participants, this is data that is not held in a routine data repository and would have to be collected on a case by case basis. Our education outcomes are robust for the point in the child’s education they refer to but they are preliminary – these children will be in school for years to come and differences we report may shift with time, disadvantages overcome for example. We do not have data for the whole cohort on language spoken at home. But we use a wide range of measures – primary care appointments and prescriptions plus outpatient and in-patient contacts with hospitals – to capture possible aetiology and degree of severity and a range of education measures that cover the first years the child is in school to capture different aspects of educational challenge. Levels of missing data are low

The results from regression models for all outcomes explored above are from multivariable models controlling for child gender, child birthweight and gestational age, mother's education status, age of mother at the birth of the child, and household means-tested benefit status. Results for all outcomes from univariable and multivariable models are not different when we account for confidence intervals around the estimated results; see Additional Analysis section 2 for full univariable and multivariable models ( Small *et al.*, 2024b). This suggests that the association between consanguinity status and poor outcomes is largely independent of other covariates that are also widely associated with poor outcomes.

## Conclusions

We have utilised cohort specific data and data collected in primary and secondary care to identify differences in mortality and in morbidity in the children of consanguineous unions. We have also looked at educational data across the years from beginning school to age 10. There are large differences in the probability of dying by age 10 years between cohort children from consanguineous and non-consanguineous unions. Children whose parents are first cousins have higher rates of primary care appointments and prescriptions. Rates of hospital events are highest for those whose parents were first cousins. Children whose parents are first cousins have higher rates of speech/ language development difficulties and learning difficulties, compared to children whose parents are not related. Turning to education data we see a similar picture, when they begin school children whose parents are first cousins are less likely to reach phonics standards and less likely to show a good level of development when compared to children whose parents are not blood relations. At age 10 there are higher numbers with special educational needs who are from first cousin unions.

## Ethics and consent

Approval for Born in Bradford was provided by Bradford Local Research Ethics committee (reference number 07/H1302/112 – approval date 1/4/2008). Research governance approval has been provided from Bradford Teaching Hospitals NHS Foundation Trust. All study participants were given Participant Information Sheets approved by the Ethics Committee before recruitment and all participants signed consent forms which included consent for data collection, usage, and data sharing.

## Data Availability

Researchers are encouraged to make use of the BiB and BiBBS data, which are available through a system of managed open access. Before you contact us, please make sure you have read our Guidance for Collaborators. Our BiB Executive reviews proposals on a monthly basis and we will endeavour to respond to your request as soon as possible. You can find out about the different datasets in our Data Dictionary. If you are unsure if we have the data that you need, please contact a member of the BiB team ( borninbradford@bthft.nhs.uk). Once you have formulated your request please complete the ‘Expression of Interest’ form available here and send to borninbradford@bthft.nhs.uk. If your request is approved we will ask you to sign a Data Sharing Contract and a Data Sharing Agreement, and if your request involves biological samples we will ask you to complete a material transfer agreement. Harvard Dataverse: Association between parental consanguinity status and child health and education outcomes, findings from the Born in Bradford cohort: Extended data. https://doi.org/10.7910/DVN/PQFSJB ( Small *et al.*, 2024b). Data are available under the terms of the Creative Commons Zero "No rights reserved" data waiver (CC0 1.0 Public domain dedication).

## References

[ref-1] Arciero E Dogra SA Malawsky DS : Fine-scale population structure and demographic history of British Pakistanis. *Nat Commun.* 2021;12(1): 7189. 10.1038/s41467-021-27394-2 34893604 PMC8664933

[ref-2] Bhopal RS Petherick ES Wright J : Potential social, economic and general health benefits of consanguineous marriage: results from the Born in Bradford cohort study. *Eur J Public Health.* 2014;24(5):862–9. 10.1093/eurpub/ckt166 24213584

[ref-3] Bishop C Small N Mason D : Improving case ascertainment of congenital anomalies: findings from a prospective birth cohort with detailed primary care record linkage. *BMJ Paediatr Open.* 2017;1(1): e000171. 10.1136/bmjpo-2017-000171 29637167 PMC5862215

[ref-4] Bishop CF Small N Parslow R : Healthcare use for children with complex needs: using routine health data linked to a multiethnic, ongoing birth cohort. *BMJ Open.* 2018;8(3): e018419. 10.1136/bmjopen-2017-018419 29525769 PMC5855244

[ref-5] Bittles AH : Consanguinity in context.Cambridge University Press,2012. 10.1017/CBO9781139015844

[ref-6] Bittles AH : Consanguineous marriages and congenital anomalies. *Lancet.* 2013;382(9901):1316–7. 10.1016/S0140-6736(13)61503-2 23830356

[ref-7] Bittles AH Black ML : The impact of consanguinity on neonatal and infant health. *Early Hum Dev.* 2010;86(11):737–41. 10.1016/j.earlhumdev.2010.08.003 20832202

[ref-8] Bittles AH Small NA : Consanguinity, genetics and definitions of kinship in the UK Pakistani population. *J Biosoc Sci.* 2016;48(6):844–854. 10.1017/S0021932015000449 26707179

[ref-9] Cheung RW Willan K Dickerson J : Risk factors for early language delay in children within a minority ethnic, bilingual, deprived environment (Born in Bradford's Better Start): a UK community birth cohort study. *BMJ Paediatr Open.* 2023;7(1): e001764. 10.1136/bmjpo-2022-001764 36927864 PMC10030670

[ref-10] Clark DW Okada Y Moore KHS : Associations of autozygosity with a broad range of human phenotypes. *Nat Commun.* 2019;10(1): 4957. 10.1038/s41467-019-12283-6 31673082 PMC6823371

[ref-11] Darr A Small N Ahmed WIU : Examining the family-centred approach to genetic testing and counselling among UK Pakistanis: a community perspective. *J Community Genet.* 2013;4(1):49–57. 10.1007/s12687-012-0117-x 23086468 PMC3537968

[ref-12] Darr A Small N Ahmed WIU : Addressing key issues in the consanguinity-related risk of autosomal recessive disorders in consanguineous communities: lessons from a qualitative study of British Pakistanis. *J Community Genet.* 2016;7(1):65–79. 10.1007/s12687-015-0252-2 26363620 PMC4715815

[ref-36] Department of Health and Social Care (DHSC): Valuing People: a new strategy for learning disability for the 21st century. *A White paper presented to Parliament by the Secretary of State for Health by Command of Her Majesty*.2001; (Accessed 13th August 2024). Reference Source

[ref-13] Gidziela A Ahmadzadeh YI Michelini G : A meta-analysis of genetic effects associated with neurodevelopmental disorders and co-occurring conditions. *Nat Hum Behav.* 2023;7(4):642–656. 10.1038/s41562-023-01530-y 36806400 PMC10129867

[ref-14] Gimenez-Lozano C Paramo-Rodriguez I Cavero-Carbonell C : Rare diseases: needs and impact for patients and families: a cross-sectional study in the Valencian Region, Spain. *Int J Environ Res Public Health.* 2022;19(16): 10366. 10.3390/ijerph191610366 36012000 PMC9408677

[ref-15] Global Burden of Disease Collaborative Network: Global burden of disease study. results. Institute for Health Metrics and Evaluation,2016. Reference Source

[ref-18] Gustavson KH : Prevalence and aetiology of congenital birth defects, infant mortality and mental retardation in Lahore, Pakistan: a prospective cohort study. *Acta Paediatr.* 2005;94(6):769–74. 10.1111/j.1651-2227.2005.tb01981.x 16188785

[ref-19] Lodh R Hou B Hough A : Health care utilisation and education outcomes of children with rare diseases: a Born in Bradford cohort study. *Eur J Pediatr.* 2023;182(12):5511–5517. 10.1007/s00431-023-05225-4 37782349

[ref-20] Malawsky DS van Walree E Jacobs BM : Influence of autozygosity on common disease risk across the phenotypic spectrum. *Cell.* 2023;186(21):4514–4527. e14. 10.1016/j.cell.2023.08.028 37757828 PMC10580289

[ref-21] Masefield S Prady S Jarvis S : The effects of caring for young children with developmental disabilities on mothers’ health and healthcare use: analysis of primary care data in the Born in Bradford cohort. *J Dev Phys Disabil.* 2022;34:67–87. 10.1007/s10882-021-09789-7

[ref-37] NIDCD (National Institute on Deafness and other Communication Disorders): Developmental language disorder.2024; (Accessed 13th August 2024). Reference Source

[ref-22] Platt L : Poverty and ethnicity in the UK.York: Joseph Rowntree Foundation,2007. Reference Source

[ref-23] Raynor P, Born in Bradford Collaborative Group: Born in Bradford, a cohort study of babies Born in Bradford, and their parents: protocol for the recruitment phase. *BMC Public Health.* 2008;8: 327. 10.1186/1471-2458-8-327 18811926 PMC2562385

[ref-24] Sheridan E Wright J Small N : Risk factors for congenital anomaly in a multiethnic birth cohort: an analysis of the Born in Bradford study. *Lancet.* 2013;382(9901):1350–9. 10.1016/S0140-6736(13)61132-0 23830354

[ref-25] Shire KA Newsham A Rahman A : Born in Bradford’s age of wonder cohort: protocol for adolescent data collection [version 1; peer review: 2 approved, 1 approved with reservations]. *Wellcome Open Res.* 2024;9:32. 10.12688/wellcomeopenres.20785.1 38770265 PMC11103777

[ref-26] Small N Bittles AH Petherick ES : Endogamy, consanguinity and the health implications of changing marital choices in the UK Pakistani community. *J Biosoc Sci.* 2017;49(4):435–446. 10.1017/S0021932016000419 27573732

[ref-28] Small N Kelly B Malawsky D : Association between parental consanguinity status and child health and education outcomes, findings from the Born in Bradford cohort: Extended data. Harvard Dataverse, V3. [Dataset].2024b. 10.7910/DVN/PQFSJB

[ref-27] Small N Kelly B Wright J : "Changes in prevalence and patterns of consanguinity in Bradford, UK - evidence from two cohort studies [version 1; peer review: awaiting peer review]. *Wellcome Open Res.* 2024a;9:222. 10.12688/wellcomeopenres.21121.1 PMC11809158 39931108

[ref-29] StataCorp: Stata statistical software: release 17.College Station, TX: StataCorp LLC,2023.

[ref-30] Tackey ND Barnes H Khambhaita P : Poverty, ethnicity and education.York: Joseph Rowntree Foundation,2011. Reference Source

[ref-31] West J Kelly B Collings PJ : Is small size at birth associated with early childhood morbidity in white British and Pakistani origin UK children aged 0–3? Findings from the Born in Bradford cohort study. *BMC Pediatr.* 2018;18(1): 22. 10.1186/s12887-018-0987-0 29390971 PMC5796403

[ref-32] Williams R : Using the margins command to estimate and interpret adjusted predictions and marginal effects. *Stata J.* 2012;12(2):308–331. 10.1177/1536867X1201200209

[ref-33] Woods CG Cox J Springell K : Quantification of homozygosity in consanguineous individuals with autosomal recessive disease. *Am J Hum Genet.* 2006;78(5):889–96. 10.1086/503875 16642444 PMC1474039

[ref-34] Wright J Small N Raynor P : Cohort profile: the Born in Bradford multi-ethnic family cohort study. *Int J Epidemiol.* 2013;42(4):978–991. 10.1093/ije/dys112 23064411

[ref-35] Zlotogora J Shalev SA : The consequences of consanguinity on the rates of malformations and major medical conditions at birth and in early childhood in inbred populations. *Am J Med Genet.* 2010;152A(8):2023–28. 10.1002/ajmg.a.33537 20635393

