# Supplementary material for: Mortality, morbidity and educational outcomes in children of consanguineous parents in the Born in Bradford cohort
Source: Wellcome Open Res. 2024 Sep 26;9:319. Originally published 2024 Jun 13. [Version 2] doi: 10.12688/wellcomeopenres.22547.2 (PMC11452767; doi:10.12688/wellcomeopenres.22547.2)
Supplement: Peer review history

# Reviewer report: Reviewer response for version 2

**DOI:** 10.21956/wellcomeopenres.25493.r101512

**Published:** 2024-10-04

**Authors:** Sajid Malik

**Affiliations:** 1 Quaid-i-Azam University, Islamabad, Pakistan

The corrections are satisfactory. The article may be accepted.

Is the work clearly and accurately presented and does it cite the current literature?

Yes

If applicable, is the statistical analysis and its interpretation appropriate?

Yes

Are all the source data underlying the results available to ensure full reproducibility?

Yes

Is the study design appropriate and is the work technically sound?

Yes

Are the conclusions drawn adequately supported by the results?

Yes

Are sufficient details of methods and analysis provided to allow replication by others?

Yes

Reviewer Expertise:

Human Genetics

I confirm that I have read this submission and believe that I have an appropriate level of expertise to confirm that it is of an acceptable scientific standard.

# Reviewer report: Reviewer response for version 2

**DOI:** 10.21956/wellcomeopenres.25493.r101513

**Published:** 2024-10-04

**Authors:** M. Mazharul Islam

**Affiliations:** 1 Department of Statistics, Sultan Qaboos University, al-Seeb, Oman

The authors have satisfactorily addressed my comments. I hereby approve the indexing of the paper.

Is the work clearly and accurately presented and does it cite the current literature?

Yes

If applicable, is the statistical analysis and its interpretation appropriate?

Yes

Are all the source data underlying the results available to ensure full reproducibility?

Yes

Is the study design appropriate and is the work technically sound?

Yes

Are the conclusions drawn adequately supported by the results?

Yes

Are sufficient details of methods and analysis provided to allow replication by others?

Yes

Reviewer Expertise:

Statistics, Demography and Public health

I confirm that I have read this submission and believe that I have an appropriate level of expertise to confirm that it is of an acceptable scientific standard.

# Reviewer report: Reviewer response for version 1

**DOI:** 10.21956/wellcomeopenres.24842.r90249

**Published:** 2024-08-22

**Authors:** Alphonse Thanaraj

**Affiliations:** 1 Dasman Diabetes Institute,, Kuwait City, Kuwait

This study is interesting as it is comprehensive to examine the relationship between consanguinity and health/education outcomes in children. The results are well-presented and well-discussed.

**I have the following comments for the authors to consider:**

- In Abstract, please indicate the cohort size, and ethnicities in methods subsection.
- It would be interesting to know whether any proportion of self-reported ‘unrelated’ were seen ‘related’ in genetically derived consanguinity.
- ‘over half (53.3%) of those who reported other blood relationships were first cousins in the genetically derived measure’ – this is quite a significant proportion wherein there is ambiguity in relatedness. This needs to be explained further and examined whether this would make big differences in the summary findings.
- Limitations from the methodologies used to assess genetically derived consanguinity can be mentioned to discuss the differences between the self-reported consanguinity and the genetically derived consanguinity.
- An examination of the observed mortality to classify the death as genetic or non-genetic can help in establishing the association between consanguinity and childhood mortality.
- A discussion on the observed rates of infant and child mortality in countries practicing heavily consanguinity, based on literature review, compared with that seen in the study cohort would be interesting.

Is the work clearly and accurately presented and does it cite the current literature?

Yes

If applicable, is the statistical analysis and its interpretation appropriate?

Yes

Are all the source data underlying the results available to ensure full reproducibility?

Partly

Is the study design appropriate and is the work technically sound?

Yes

Are the conclusions drawn adequately supported by the results?

Yes

Are sufficient details of methods and analysis provided to allow replication by others?

Yes

Reviewer Expertise:

Genetic epidemiology

I confirm that I have read this submission and believe that I have an appropriate level of expertise to confirm that it is of an acceptable scientific standard, however I have significant reservations, as outlined above.

# Response

**Published:** 2024-09-18

**Authors:** Neil Small

**Affiliations:** Faculty of Health Studies, University of Bradford, Bradford, England, UK

We are grateful for the expertise our reviewers have provided in commenting on our paper and for the opportunity to address their concerns and questions. We hope the following responses do this.

 1. In Abstract, please indicate the cohort size, and ethnicities in methods subsection. We have added to the abstract in the ‘Methods’ section which now includes:  

**Response-**The cohort comprises of 13727 children; 35.7% of whom are White British, 43.7% are Pakistani heritage, and 20.8% are from other ethnic groups.”

 2.It would be interesting to know whether any proportion of self-reported ‘unrelated’ were seen ‘related’ in genetically derived consanguinity.

**Response-**We do show this in the extended data – in table S3.2 8.5% of those who self-reported as unrelated were deemed to be related in the genetically derived consanguinity status.  

 3.‘over half (53.3%) of those who reported other blood relationships were first cousins in the genetically derived measure’ – this is quite a significant proportion wherein there is ambiguity in relatedness. This needs to be explained further and examined whether this would make big differences in the summary findings.

**Response-** In the final paragraph in section “4.1 Sensitivity analysis using genetically derived consanguinity status for a subset of children” we do address this. The text in this section of the paper is: “We re-ran regression models for differences in the health and education outcomes using the genetically derived consanguinity status measure, see Additional Analysis 4 ( Small et al., 2024b). The differences in health and educational outcomes by consanguinity status were generally very similar whether the measure of consanguinity status was self-reported or genetically derived. However, there are differences between the self-reported and genetically derived measures of the probability of dying by the age of 10 years. As noted above there was more missing data for the genetically derived measure for children who have died. This is likely to explain differences in the probabilities of dying between the self-report and genetically derived.”   To make this clearer we have added a few words to the first sentence of the above to say **“Due to the differences in consanguinity status of parents between the self-reported and genetically derived measures we re-ran regression models for differences in the health and education outcomes…”**

 4.Limitations from the methodologies used to assess genetically derived consanguinity can be mentioned to discuss the differences between the self-reported consanguinity and the genetically derived consanguinity.

**Response-  **In the ‘Discussion’ section we do discuss this in some detail, see the “On the sensitivity analysis” part of the discussion. “We have reported genetically derived consanguinity data for a subset of the BiB cohort and found a close match between self-identified and genetically identified first cousins but differences in those who self-identify as being in “other blood” relationships. Over half of those couples describing themselves as related but not first cousins appear to be first cousins on genetic analysis. This disparity may be a result of a lack of clarity in interviewees about what a first cousin and what “other-blood” is, it may be to do with people answering “other-blood” because they see a stigma attached to cousin-marriage and are seeking to mitigate this in the self-description they report to researchers. Sheridan et al. (2013) reported higher rates of CAs in the children of other blood unions (then identified through self-reporting) than were expected from a formal calculation of the relationship coefficient, a measure of genetic closeness (Sheridan et al., 2013: 8). This might be to do with endogamy, a longstanding tradition of consanguinity in a specific population allied with population stratification in marriage choices (see Bittles & Small, 2016; Small et al., 2017; Woods et al., 2006; Zlotogora & Shalev, 2010). In effect one can be akin to a cousin genetically, even if one is not a cousin in the familial sense. We will report separately on a qualitative study in Bradford contemporaneous with this one seeking views on the current importance of consanguinity in peoples’ choices of marriage partner.   We could assume that the genetically derived consanguinity measures would have less measurement error than the self-reported measures, therefore the size of observed effects would be larger. However, the results we report were essentially the same when using both measures apart from cases where the children with missing data were different. Given that children who have genetically derived data in BiB exclude many who died or were pre-term births, it may be that they could be considered as distinctly different samples. Therefore, the similarity of results using both measures could be seen as evidence of the robustness of the findings presented.”   We are not sure what else we could add.

 5. An examination of the observed mortality to classify the death as genetic or non-genetic can help in establishing the association between consanguinity and childhood mortality.

**Response-**Unfortunately, we do not have data on cause of death. However, the number of children who died was small, only 172. The reference below is to a paper that found 22% of infant deaths were due to genetic disorders – that would suggest around 38 of the child deaths in the BiB cohort may be due to genetic disorders. So, even if we did have cause of death, the numbers would be too small for regression analysis.   Wojcik MH, Schwartz TS, Thiele KE, Paterson H, Stadelmaier R, Mullen TE, VanNoy GE, Genetti CA, Madden JA, Gubbels CS, Yu TW, Tan WH, Agrawal PB. Infant mortality: the contribution of genetic disorders. J Perinatol. 2019 Dec;39(12):1611-1619.    

 6.A discussion on the observed rates of infant and child mortality in countries practicing heavily consanguinity, based on literature review, compared with that seen in the study cohort would be interesting.

**Response**-If we look at observed rates of infant and child mortality in countries with high levels of consanguinity we see rates varying greatly according to levels of poverty and access to healthcare provision. The text below provides some detail, and the references cited give access to detailed data which underlines this text.   We have added to the article in the first section of our Discussion the following: **“There are 32 countries in the world with consanguinity rates above 5% (according to The World Population Review -** **https://worldpopulationreview.com/country-rankings/inbreeding-by-country** **)** **.** **The under 5 mortality rate for these countries ranges from 3.4 to 107.2 per 1,000 live births (according to the World Bank -** **https://data.worldbank.org/indicator/SH.DYN.MORT** **). Some countries with similar rates of consanguinity have vastly different under 5 mortality rates; for example, in the United Arab Emirates 50.5% of marriages are consanguineous and the under 5 mortality rate is 5.3 per 1,000 live births, while in South Sudan 50.0% of marriages are consanguineous and the under 5 mortality rate is 98.8 per 1,000 live births. Similarly in Tunisia 21.1% of marriages are consanguineous and the under 5 mortality rate is 11.5 per 1,000 live births, while in Nigeria 19.9% of marriages are consanguineous and the under 5 mortality rate is 107.2 per 1,000 live births.  Countries vary greatly on levels of poverty and on the availability of healthcare provision, and these are the factors that impact most on child death rates.”**

# Reviewer report: Reviewer response for version 1

**DOI:** 10.21956/wellcomeopenres.24842.r87028

**Published:** 2024-08-09

**Authors:** M. Mazharul Islam

**Affiliations:** 1 Department of Statistics, Sultan Qaboos University, al-Seeb, Oman

This study explores the link between a parent's consanguinity and a child's health and education outcomes. It is notable that it is grounded in a prospective analysis of data from a UK-based longitudinal family cohort known as "Born in Bradford." The paper is well-written and has a sound methodology. However, I have a few observations that need clarification for further improvement.

  

1. It appears that all the dependent and independent variables are involved with huge missing values. In univariate analysis (Tables 1, 2, and 3), the authors considered valid percentages, ignoring the missing values. However, in multivariable analysis (Table 4), it is not clear how the authors handle the missing values. For an outcome variable, when it was dichotomized, did the authors consider the missing value as 0?
2. It would be better to identify the significant covariates of consanguinity by employing the Chi-square test in Table 2.
3. Under ‘Plan of analysis’ the authors simply mentioned that the logistic regression model was used for the dichotomous outcome variables and Poisson regression for the count variables without specifying the variables. 

Is the work clearly and accurately presented and does it cite the current literature?

Yes

If applicable, is the statistical analysis and its interpretation appropriate?

Yes

Are all the source data underlying the results available to ensure full reproducibility?

Yes

Is the study design appropriate and is the work technically sound?

Yes

Are the conclusions drawn adequately supported by the results?

Yes

Are sufficient details of methods and analysis provided to allow replication by others?

Yes

Reviewer Expertise:

Statistics, Demography and Public health

I confirm that I have read this submission and believe that I have an appropriate level of expertise to confirm that it is of an acceptable scientific standard, however I have significant reservations, as outlined above.

# Response

**Published:** 2024-09-18

**Authors:** Neil Small

**Affiliations:** Faculty of Health Studies, University of Bradford, Bradford, England, UK

We are grateful for the expertise our reviewers have provided in commenting on our paper and for the opportunity to address their concerns and questions. We hope the following responses do this.

 1.It appears that all the dependent and independent variables are involved with huge missing values. In univariate analysis (Tables 1, 2, and 3), the authors considered valid percentages, ignoring the missing values. However, in multivariable analysis (Table 4), it is not clear how the authors handle the missing values. For an outcome variable, when it was dichotomized, did the authors consider the missing value as 0?

**Response-**As noted in the paper, and outlined in Tables 1, 3a, 3b, there is a degree of missing data. However not all dependent and independent variables have huge amounts of missing data. For the outcome (dependent variables) there is no missing data on the probability of dying by age 10 years, less than 5% missing (636 cases missing of the 13,727 children) for the outcomes from routine health data (primary care appointments, primary care prescriptions, hospital accident and emergency events, hospital outpatient events, diagnosis of learning difficulties, and diagnosis of speech and language difficulties). There is more missing data on educational outcomes, around 19%, as not all children had linked educational data. On the independent variables there were varying amounts of missing data. Those measures derived from routine data (gender, birthweight, gestational age) have a very small missing data, ranging from 1.27% to 3.6%. Those measures derived from the baseline questionnaire have the highest levels of missing data, over 20% missing. This is due to not all women completing a baseline questionnaire.   We feel that the paper, tables and text, notes the levels of missing data. But we very much welcome the reviewer noting that we have not made it clear how we dealt with missing data. Therefore, we have added some additional text at the end of the ‘plan of analysis section’ to address this omission. **“In all regression models we dealt with missing data by carrying out a complete case analysis.”**

 2.It would be better to identify the significant covariates of consanguinity by employing the Chi-square test in Table 2.

**Response**- **We have added p values derived from Chi-squared tests to Table 2**. Two of our reviewers requested that we add p-values and we have accommodated this. We did not include them in our initial submission because we were persuaded by the American Statistical Association “Statement on Statistical Significance and *P*-values” with six principles. Principle 6 is : A P-value, or statistical significance, does not measure the size of an effect or the importance of a result. The threshold of statistical significance that is commonly used is a *P*-value of 0.05. This is conventional and arbitrary. It does not convey any meaningful evidence of the size of the effect. See:  Yaddanapudi LN. The American Statistical Association statement on *P*-values explained. J Anaesthesiol Clin Pharmacol. 2016 Oct-Dec;32(4):421-423. doi: 10.4103/0970-9185.194772. PMID: 28096569; PMCID: PMC5187603.

 3. Under ‘Plan of analysis’ the authors simply mentioned that the logistic regression model was used for the dichotomous outcome variables and Poisson regression for the count variables without specifying the variables. 

**Response-**We have added some additional text to the first paragraph of the ‘Plan of analysis’ section, to make it clear which outcomes are considered in the logistic and Poisson regression models. The relevant text now reads: **“We employed logistic regression for dichotomous outcomes (whether the child died, diagnosis of learning difficulties, diagnosis of speech and language difficulties, reaching good stage of development in school reception year, achieving phonics standard by school year 1, being recorded as having special educational need by age 10 years), and Poisson regression for counts of healthcare use events (primary care appointments, primary care prescriptions, hospital accident and emergency events, hospital outpatient events). We estimated odds ratios for dichotomous outcomes and incident rate ratios for counts of events.”**

# Reviewer report: Reviewer response for version 1

**DOI:** 10.21956/wellcomeopenres.24842.r87020

**Published:** 2024-07-08

**Authors:** Sajid Malik

**Affiliations:** 1 Quaid-i-Azam University, Islamabad, Pakistan

Mortality, morbidity and educational outcomes in children of consanguineous parents in the Born in Bradford cohort

 Small N, Kelly B, Malawsky DS, Lodh R, Oddie S and Wright J

 In this study, Small et al. employ a prospective cohort of Born in Bradford and assess the differentials according to the consanguinity status of children in mortality, health care usage, health and educational outcomes. This is an interesting study, however, the manuscript would benefit from the following corrections:

  

1. 'Other blood relations' needs to be elaborated, as there are several consanguineous marriage types other than first cousins.
2. The basic demographic information of the cohort is missing, including origin, ethnicity, language, etc. It is very important to understand the distribution of consanguinity across the ethnic groups.  
3. While there is sufficient detail given for educational data, the detailed definitions of birth outcome are missing in the Methods section.
4. Methods: Please elaborate how incident rate ratios were calculated.  
5. Table 2: It is worthwhile to compare the columns and provide significance of difference by employing Chi-square test.
6. Sensitivity analysis using genetically derived consanguinity status for a subset of children: The authors re-utilized the previous data for sensitivity analyses. It is worthwhile to give the composition of major ethnicities employed in the homozygosity analyses.

Is the work clearly and accurately presented and does it cite the current literature?

Yes

If applicable, is the statistical analysis and its interpretation appropriate?

Yes

Are all the source data underlying the results available to ensure full reproducibility?

Yes

Is the study design appropriate and is the work technically sound?

Yes

Are the conclusions drawn adequately supported by the results?

Yes

Are sufficient details of methods and analysis provided to allow replication by others?

Yes

Reviewer Expertise:

Human Genetics

I confirm that I have read this submission and believe that I have an appropriate level of expertise to confirm that it is of an acceptable scientific standard, however I have significant reservations, as outlined above.

# Response

**Published:** 2024-09-18

**Authors:** Neil Small

**Affiliations:** Faculty of Health Studies, University of Bradford, Bradford, England, UK

We are grateful for the expertise our reviewers have provided in commenting on our paper and for the opportunity to address their concerns and questions. We hope the following responses do this.

1. 'Other blood relations' needs to be elaborated, as there are several consanguineous marriage types other than first cousins.
**Response: **We have expanded the first paragraph in the ‘Data: consanguinity exposure measure and other covariates’ section; it now reads: “Self-reported consanguinity status was collected as part of a wide-ranging interviewer administered questionnaire at recruitment to BiB. A section of this questionnaire asked whether the woman was related to the father of their baby. If they answered “yes” they were then asked in what way they were related; **with the options in the questionnaire being** **‘First cousin’,** **‘First cousin once removed’, ‘Second cousin’ and ‘Other related by blood’**. The answers to these two questions were used to construct three categories of consanguinity; children whose parents were not blood related (‘not related’), children whose parents were first cousins (‘first cousins’), and children whose parents were other blood relations (‘other blood relations’).” The ‘Other blood relations’ category used in the analysis is comprised of ‘First cousin once removed’, ‘Second cousin’ and ‘Other related by blood’.” The text in bold identifies the addition we have made to this paragraph.
2. The basic demographic information of the cohort is missing, including origin, ethnicity, language, etc. It is very important to understand the distribution of consanguinity across the ethnic groups.  
**Response:** We have added child ethnicity to Table 1 and to Table 2. We have also added details of Mother’s country of birth to Table 2.  To respond to the request for details of ‘origin’ we have added the following sentence to the text “ **The majority of mothers in the study were born in England (7038: 62.5%), 2887 mothers (25.6%) were born in Pakistan. The remainder were born in a wide range of countries with no one country providing more than 1.5% of mothers.”**  All the children are born in Bradford, so are English/ British; they have attended school in Bradford and hence all children speak English (we do not have data on other languages spoken at home). We have also added the following sentence: **“In our study population, recruited between 2007 and 2010, over 90% of children whose parents were first cousins or other blood relations were of Pakistani heritage. Rates of consanguinity amongst the population in Bradford have fallen substantially over recent years (Small et all 2024a).”**  
3. While there is sufficient detail given for educational data, the detailed definitions of birth outcome are missing in the Methods section.
**Response: **We feel we have adequately described most of the non-educational outcomes, though your comment has led us to realise we should give more detail of the outcomes of diagnosis of learning difficulties, and the diagnosis of speech and language difficulties, by age 10 years. To expand on the first point: we feel the outcome of death by the age of 10 years needs no further definition. The outcomes of counts of primary care appointments, primary care prescriptions, hospital accident and emergency events, and hospital outpatient events are simply defined by counts of these events. And in the additional analysis we list the Read codes that were used to identify hospital accident and emergency, and hospital outpatient events (Table S1.1: Codes identified from a text search, classified as accident and emergency of outpatient hospital events).   On the second point, regarding the need for additional definition of the outcomes of diagnosis of learning difficulties, and the diagnosis of speech and language difficulties, by age 10 years. Although we give the list of Read codes that were used to identify such diagnosis in the additional analysis, we have added some additional text to give the reader a better understanding of these conditions.   Therefore, we have added additional text to the section “Data: outcomes” after we mention learning difficulties we have added: **“** **A learning difficulty is defined by the Department of Health and Social Care (DHSC) as: “a significantly reduced ability to understand new or complex information, to learn new skills (impaired intelligence), with a reduced ability to cope independently (impaired social functioning), which started before adulthood” (DHSC 2001)”**   And after we mention speech and language difficulties we have added: **“Developmental language disorder is a communication disorder that interferes with learning, understanding, and using language. These language difficulties are not explained by other conditions, such as hearing loss or autism, or by extenuating circumstances, such as lack of exposure to language (NIDCD 2024).” **We have also details of the two references included in this additional material: Department of Health and Social Care (DHSC) 2001 and NIDCD 2024.  
4. Methods: Please elaborate how incident rate ratios were calculated. **Response: **We state in the ‘Plan of analysis section’ that we used Poisson regression models to estimate counts of events (primary care appointments, primary care prescriptions, hospital accident and emergency events, and hospital outpatient events). These counts are illustrated as rates per year for primary care outcomes, and rates for the ten year period for hospital events in Figure 2, with the actual values reported in the additional analysis.   Regardless of whether they are counts per year or counts per ten years the incident rate ratios are simply the ratio of rates for one group divided by the ratio of rates for the reference group. As we understand it there is only one way to calculate incident rate ratios, and that was what we did (it is simply a ratio of two rates, like an odds ratio is simply a ratio of two sets of odds).   For details of Poisson regression and the calculation of incident rate ratios see: https://www.stata.com/manuals13/rpoisson.pdf At the bottom of page 3 the formula for deriving incident rate ratios is given.   So, incident rate ratios are the incident rate of the group of interest divided by the incident rate of the reference group (often expressed as the incident rate of the exposed group divided by the incident rate of the control group).
5. Table 2: It is worthwhile to compare the columns and provide significance of difference by employing Chi-square test.
**Response:** **We have added p values derived from Chi-squared tests to Table 2.** Two of our reviewers requested that we add p-values and we have accommodated this. We did not include them in our initial submission because we were persuaded by the American Statistical Association “Statement on Statistical Significance and *P*-values” with six principles. Principle 6 is : A P-value, or statistical significance, does not measure the size of an effect or the importance of a result. The threshold of statistical significance that is commonly used is a *P*-value of 0.05. This is conventional and arbitrary. It does not convey any meaningful evidence of the size of the effect. See:  Yaddanapudi LN. The American Statistical Association statement on *P*-values explained. J Anaesthesiol Clin Pharmacol. 2016 Oct-Dec;32(4):421-423. doi: 10.4103/0970-9185.194772. PMID: 28096569; PMCID: PMC5187603.
6. Sensitivity analysis using genetically derived consanguinity status for a subset of children: The authors re-utilized the previous data for sensitivity analyses. It is worthwhile to give the composition of major ethnicities employed in the homozygosity analyses.
**Response: **We have added some additional text to the section 4.1 Sensitivity analysis using genetically derived consanguinity status for a subset of children’ to give details of the ethnic composition of the children with genetic data. “ **The ethnicity of the subset of children who had genetically derived consanguinity status was very similar to the ethnicity of the children in the sample using self-reported consanguinity. Just over a third (35.6%) were White British, 44.1% were Pakistani heritage, and 20.3% were from other ethnic groups.”**
